# Supplementary material for: Early-warning signals for Dansgaard-Oeschger events in a high-resolution ice core record
Source: Nat Commun. 2018 Jul 2;9:2556. doi: 10.1038/s41467-018-04881-7 (PMC6028441; doi:10.1038/s41467-018-04881-7)
Supplement: Supplementary file 1 — Supplementary Information [file 41467_2018_4881_MOESM1_ESM.pdf]

Supplementary Information for "Early-warning signals for Dansgaard-Oeschger events in a high-resolution ice core record"

Niklas Boers[1,2*]

[1]Grantham Institute for Climate Change, Imperial College, London, UK
[2]Potsdam Institute for Climate Impact Research, Potsdam, Germany.
[*]E-mail: nboers@ic.ac.uk and boers@pik-potsdam.de

Supplementary Tables

Supplementary Table 1: **Beginning and end dates of the intervals in which EWS are searched for.** Beginning and end dates of the Greenland interstadial (GI) intervals in accordance with [1], but for the higher-resolution time series used here. The dates are determined using the methodology described in [2], where it was shown that they are in close agreement with the visual identification of [3]. The first line (numbered 0) is for the Younger-Dryas/Preboreal transition, which is not a DO event.

#	beginning (yr b2k)	end (yr b2k)
0	12 900	11 705
1	23 105	14 690
2	27 460	23 375
3	28 510	27 790
4	32 025	28 910
5	33 390	32 520
6	34 725	33 735
7	36 590	35 505
8	39 935	38 235
9	40 790	40 165
10	42 100	41 480
11	44 285	43 365
12	49 105	46 860
13	51 650	49 315
14	54 745	54 235
15	56 440	55 815
16	58 515	58 280

Supplementary Figures

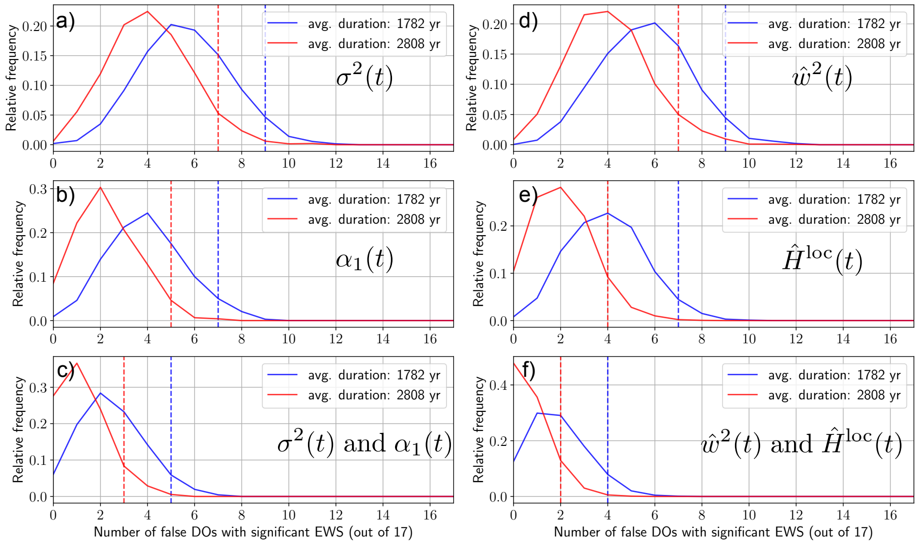

Supplementary Figure 1: **Null-model distributions for the number of early-warning signals (EWS) in the different estimators for the δ^{18}O time series.** a) Null-model distribution for the number of EWS in σ^2, b) Null-model distribution for the number of EWS in α_1, c) Null-model distribution for simultaneous EWS in both sσ^2 and α_1. d–f) Same as (a–c), but for the wavelet-based estimators $\hat{w}^2$ and $\hat{H}^{\mathrm{loc}}$. The blue lines show the null model distributions obtained when only the Greenland stadial (GS) intervals are considered (with average duration of 1782 yr), and the red lines show the null model distributions when EWS are searched for during the entire intervals between subsequent Dansgaard-Oeschger (DO) events (with average duration of 2808 yr). In all panels, the vertical dashed lines mark the respective significance thresholds at 95% confidence level, which are also given in Tab. 1 of the main text (see Methods for further details).

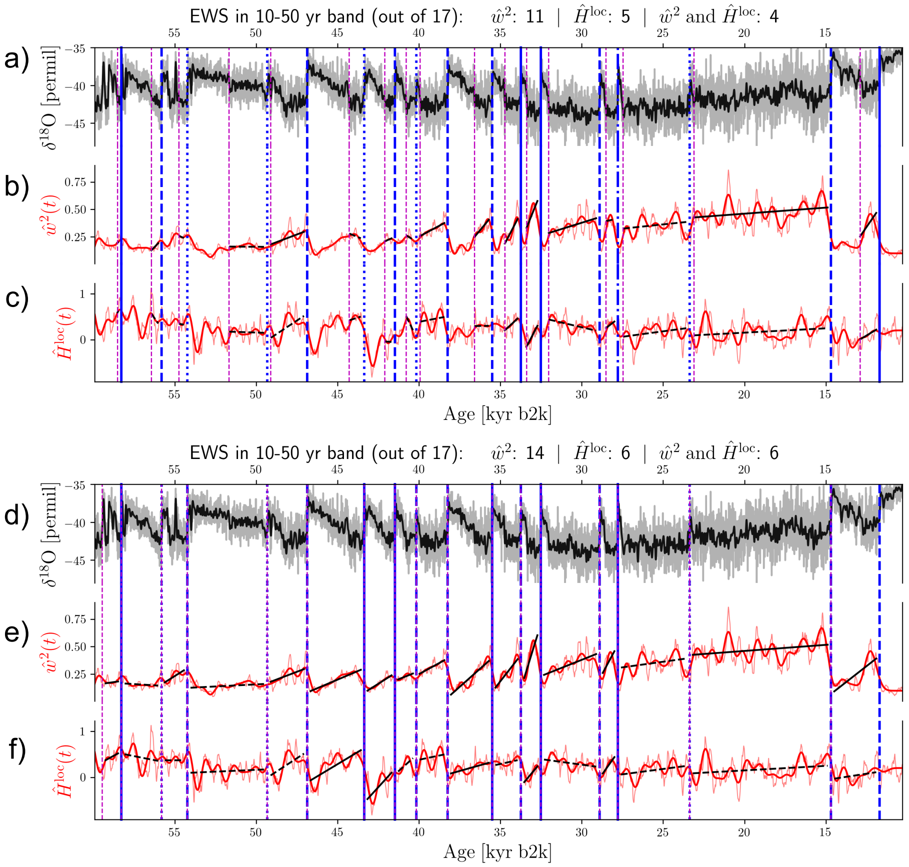

Supplementary Figure 2: **Same as Fig. 3 in the main text, but using the Morlet instead of the Paul wavelet basis.** a) The 5-yr-interpolated δ^{18}O time series (grey), together with a low-pass filtered version (black) for visual clarity. Dansgaard-Oeschger (DO) events, as well as the Younger-Dryas/Preboreal transition, are marked by the vertical blue lines, and the preceding ends of Greenland interstadial (GI) intervals by vertical dashed magenta lines (cf. Supplementary Table 1 for corresponding ages). Early-warning signals (EWS) are searched for in the Greenland stadial (GS) intervals between the magenta and blue lines. b) Time series of the wavelet-based estimator of high-frequency fluctuations $\hat{w}^2$, confined to the 10–50-yr periodicity band (red, see text for details). c) Same as (b), but for the local Hurst exponent $\hat{H}^{loc}$. d–f) Same as (a–c), but here, EWS are searched for in the entire intervals between subsequent DO transitions. Significant (non-significant) trends according to the phase-randomization test (see Methods) are indicated by the solid (dashed) black lines. Transition events that are preceded by significant EWS in $\hat{w}^2$ ($\hat{H}^{loc}$) are marked by dashed (dash-dotted) blue lines, and transitions that are preceded by EWS in both $\hat{w}^2$ and $\hat{H}^{loc}$ are indicated by solid blue lines. If neither of the two estimators shows significant EWS, the blue lines are dotted.

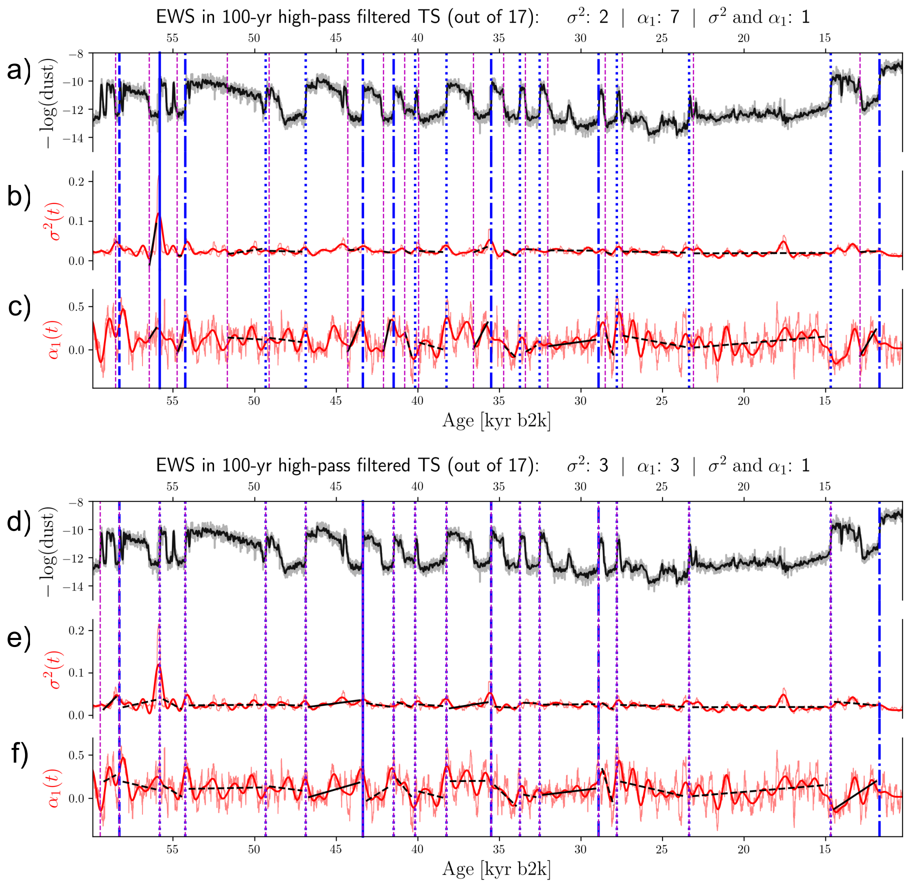

Supplementary Figure 3: **Same as Fig. 2 in the main text, but for the dust time series.** a) The 5-yr-interpolated dust time series (grey), together with a low-pass filtered version (black) for visual clarity. Dansgaard-Oeschger (DO) events, as well as the Younger-Dryas/Preboreal transition, are marked by the vertical blue lines, and the preceding ends of Greenland interstadial (GI) intervals by vertical dashed magenta lines (cf. Supplementary Table 1 for corresponding ages). Early-warning signals (EWS) are searched for in the Greenland stadial (GS) intervals between the magenta and blue lines. b) Time series of the variance σ^2 of the 100-yr high-pass filtered dust time series (red, see text for details). c) Same as (b), but for the lag-1 autocorrelation coefficient α_1. d–f) Same as (a–c), but here, EWS are searched for in the entire intervals between subsequent DO transitions. Significant (non-significant) trends according to the phase-randomization test (see Methods) are indicated by the solid (dashed) black lines. Transition events that are preceded by significant EWS in σ^2 (α_1) are marked by dashed (dash-dotted) blue lines, and transitions that are preceded by EWS in both σ^2 and α_1 are indicated by solid blue lines. If neither of the two estimators shows significant EWS, the blue lines are dotted.

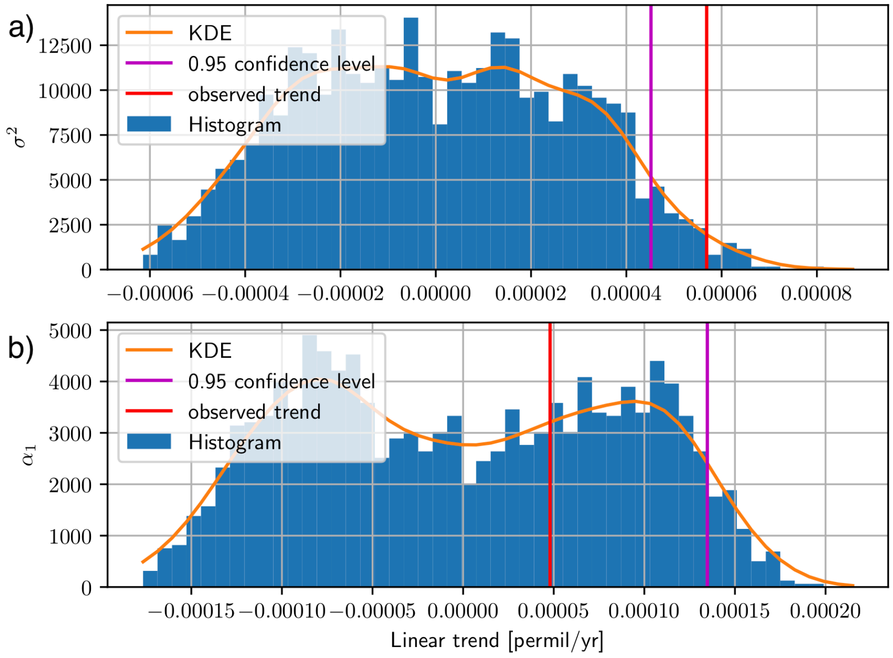

Supplementary Figure 4: **Null-model distribution for linear trends in early-warning signal estimators prior to Dansgaard-Oeschger event 1.** a) Histogram (blue bars) and kernel density estimate of the probability density function (orange line) of the null model distribution for the linear trend of $\sigma^2(t)$ in the GS prior to Dansgaard-Oeschger event 1 (DO-1). The null model distribution is obtained from surrogates constructed by randomizing the phases of the observed, detrended $\sigma^2(t)$ in the corresponding Greenland stadial (GS) interval (see Methods). The magenta vertical line shows the 0.95 confidence level, the red vertical line the position of the observed trend. b) Same as (a), but for the autocorrelation coefficient α_1. Corresponding distributions are constructed for all early-warning signal (EWS) estimators and GS, as well as GS-and-GI intervals.

References

[1] Rypdal, M. Early-Warning Signals for the onsets of Greenland Interstadials and the Younger Dryas-Preboreal transition. *Journal of Climate* JCLI–D–15–0828.1 (2016). URL http://journals.ametsoc.org/doi/10.1175/JCLI-D-15-0828.1.

[2] Rousseau, D. D. *et al.* (MIS3 & 2) millennial oscillations in Greenland dust and Eurasian aeolian records ?? A paleosol perspective. *Quaternary Science Reviews* **169**, 99–113 (2017).

[3] Rasmussen, S. O. *et al.* A stratigraphic framework for abrupt climatic changes during the Last Glacial period based on three synchronized Greenland ice-core records: Refining and extending the INTIMATE event stratigraphy. *Quaternary Science Reviews* **106**, 14–28 (2014). URL http://dx.doi.org/10.1016/j.quascirev.2014.09.007.
